# The Effects of Introducing a Harm Threshold for Medical Treatment Decisions for Children in the Courts of England & Wales: An (Inter)National Case Law Analysis

**DOI:** 10.1007/s10728-023-00472-w

**Published:** 2023-12-18

**Authors:** Veronica M. E. Neefjes

**Affiliations:** Centre for Social Ethics and Policy, School of Law, University of Manchester, Oxford Road, Manchester, M13 9PL UK

**Keywords:** Harm threshold, Best interests test, Children, Parental decision-making, Medical treatment, Case law

## Abstract

The case of Charlie Gard sparked an ongoing public and academic debate whether in court decisions about medical treatment for children in England & Wales the best interests test should be replaced by a harm threshold. However, the literature has scantly considered (1) what the impact of such a replacement would be on future litigation and (2) how a harm threshold should be introduced: for triage or as standard for decision-making. This article directly addresses these gaps, by first analysing reported cases in England & Wales about medical treatment in the context of a S31 order, thus using a harm threshold for triage and second comparing court decisions about medical treatment for children in England & Wales based on the best interest test with Dutch and German case law using a harm threshold. The investigation found that whilst no substantial increase of parental discretion can be expected an introduction of a harm threshold for triage would change litigation. In particular, cases in which harm is limited, currently only heard when there are concerns about parental decision-making, may be denied a court hearing as might cases in which the child has lost their capacity to suffer. Applying a harm threshold for triage in decisions about withholding or withdrawing life-sustaining treatment might lead to a continuation of medical treatment that could be considered futile.

**Supplementary Information:**

The online version contains supplementary material available at 10.1007/s10728-023-00472-w.

## Introduction

The court cases of Alfie Evans and Charlie Gard [1, 27] that decided the withdrawal of their life-sustaining treatment drew world-wide attention. Interventions from the US President [40], the Pope [13], and an ‘army’ of supporters storming the hospital [12] turned usually private disagreements between parents and clinicians into global spectacles.

Whilst conflicts about medical treatment for seriously ill children have been litigated in the courts of England & Wales for many years, following these high profile cases parents [66] and academics [18, 36, 65] have argued for a new approach; in particular, for the replacement of the best interests test by a harm threshold. This argument has gained traction following the appeal by the legal team representing Charlie Gard’s parents, who proposed that when alternative medical treatment is available parental decision making should prevail *unless* their decision causes significant harm to the child[38].

In academia several arguments have been put forward in favour of an introduction of a harm threshold. Some relate to criticism of the best interests test, i.e. that it is ill-defined or unreasonably demanding [22, 31, 63], claims I do not discuss here. Others strive for different outcomes, such as increased parental discretion [63, 66] and a reduction of cases decided in court [43]. However, what is lacking from the discussion of outcomes is *evidence* about what the effects of introducing a harm threshold in the courts of England & Wales would be.

Section 31(2) of the Children Act 1989 (hereafter referred to as the Children Act) contains a harm threshold that must be crossed before a care or supervision (S31) order can be made. The aim of including the harm threshold in the Children Act was *explicitly* to safeguard parents from unwarranted State intervention. According to now Lady Hale the harm threshold was ‘designed to restrict compulsory intervention to cases which genuinely warrant it’ [37]

Whether the harm threshold in the Children Act has achieved its goal is difficult to say. In England, the proportion of children subject to S31 orders continues to rise despite its enactment. Their number has more than doubled between 2007 and 2017 [34] which might suggest that the harm threshold is not sufficiently protective. However, many factors may contribute to the rise of S31 orders of which the extent of protection offered by the harm threshold is only one.

Amongst those in favour of introducing a harm threshold there is some disagreement about *how* a harm threshold should be introduced. In England & Wales court decisions about children are a two-step process; the first step is a triage decision, that answers the question *whether* the case can be heard in court, and the second the actual court decision [19]. Some argue for the replacement of the best interests test by a harm threshold for triage [25] whilst others argue for a replacement of the standard to be applied in decision-making [21]. The distinction is important because triage tests by their nature are ‘rough and quick’ [25] whereas a substantive determination whether the threshold is crossed ideally involves a thorough and holistic assessment.

In view of recent clinical, legal, and academic developments, this paper aims to provide much needed insight about the expected effects of introducing a harm threshold in the courts of England & Wales either for triage or as standard for decision-making. To do so, I analyse and compare case law regarding medical treatment decisions for children in England & Wales with case law in the Netherlands and Germany, two jurisdictions that use a harm threshold. Based on the investigation, I conclude that replacing the best interests test for a harm threshold is unlikely to increase parental discretion or reduce the number of court cases but will introduce new challenges.

The article starts with a short description of the methodology followed by an analysis of the legal context in the three jurisdictions in which the harm threshold operates. This analysis generated two main findings. First, the lack of a legal equivalence of inherent jurisdiction in the Netherlands and Germany determines that only cases in which parents refuse medical treatment can be litigated. Second, only courts in England & Wales scrutinise parental care against an objective standard *in addition* to evaluating the significance of harm to the child. The latter finding is confirmed in the subsequent analysis of medical treatment decisions in the context of S31 procedures. The analysis and comparison of national and international case law finds that triage decisions using a harm threshold might prevent litigation of cases in which the harm is limited but because of their rarity the effect thereof will be small; these cases currently only reach the courts when there are concerns about parental decision-making. When the harm threshold is used as standard for decision-making the outcome of cases will remain largely unchanged. An application of the findings to decisions about withholding and withdrawing life-sustaining treatment in England & Wales concludes that when the harm threshold is used as a standard for decision-making the outcome of cases will likely not change. However, introducing the harm threshold for triage could prevent cases reaching the court when harm is limited because the child has lost their ability to suffer. The harm threshold introduced for triage may increase the number of children continuing on life-sustaining treatment considered futile by their clinicians.

## Methods

The investigation analyses both national case law about medical treatment decisions for children in the context of S31 orders, thus using a harm threshold for triage and functionally compares [35] national and international case law, thus best interests decisions with harm threshold decisions. The comparator jurisdictions, the Netherlands and Germany, are suitable because both have used a harm threshold for decisions about children for more than a century and their societies are broadly similar to England & Wales with regards to views on medical ethics and diversity of the population. The structural difference, the Netherlands and Germany are under Roman Law whilst England & Wales operate a common law system is of minor importance in this context. Decisions regarding children in the three jurisdictions rely on statutory law and in England & Wales case law ruling medical treatment decisions for children in court is now well settled [28].

For England & Wales cases were identified by a search of the legal databases Lexis Library (www.lexisnexis.com) and BAILII (www.bailii.org) with the search terms ‘medical treatment’, ‘child’ and ‘minor’. For the Netherlands www.rechtspraak.nl and for Germany www.rechtsportal.de and FamRz (www.gieseking-digital.de) were searched with the same search terms in Dutch and German respectively. Searches were last performed in July 2022. Judgments were eligible for inclusion when (1) it decided a dispute regarding medical treatment between parents and medical professionals, (2) the case was heard between 1st January 1990 and 1st July 2022 and (3) the parents (not the child) were the family decision makers. Notably, in the Netherlands and Germany clinicians or hospitals cannot directly apply to the courts but must alert their local child protection organisation that then takes on responsibility for litigation. The extent of clinician’s involvement in litigation can thus be slightly more ambiguous than in England & Wales.

## Comparing Jurisdictions

### Legal Context

The most important difference in legal context between England & Wales on the one hand and Germany and the Netherlands on the other is the lack of a legal equivalent of the inherent jurisdiction in the latter jurisdictions. In England & Wales the powers of the court are derived from two sources; statutory power from parliament and the inherent jurisdiction originating in the duties of the Crown to protect its citizens [30]; the power of the courts in Germany and the Netherlands is derived from statutory law only.

With the statutory powers as outlined in the Children Act the courts can issue a so-called Sect. 8 order which usually takes the form of a specific issue order. With a specific issue order the court can either prohibit or give consent for specific medical treatment. The inherent jurisdiction also allows the courts to make declarations, i.e. that a treatment proposal is lawful and/or in the best interests of a child [30]. Similar to England & Wales, courts in the Netherlands and Germany can prohibit or give substituted consent for medical treatment. However, there is no statutory law in place in either jurisdiction that allows courts to make declarations about the lawfulness of a particular treatment proposal for children. Neither clinicians nor parents can approach the courts to arbitrate a conflict about a proposal to withdraw or withhold life-sustaining treatment *unless* the parental decision can be framed as exposing the child to significant harm.

The lack of a legal equivalent of the inherent jurisdiction in the Netherlands and Germany is important because it determines the type of case that can be arbitrated in court.

The courts in England & Wales decide about withholding and withdrawing medical treatment and parental refusals of medical treatment in almost equal numbers [48] whereas reported Dutch and German cases are about parental refusal of medical treatment or, rarely, about prohibition of intended treatment.

In summary, the three jurisdictions are alike in that they can rely on their courts for resolution of conflicts about parental *refusal* of medical treatment. Where parents and clinicians disagree regarding *withholding* or *withdrawing* treatment neither the Dutch nor German courts can decide unless the parental decision crosses the harm threshold.

### Comparing Harm Thresholds

Section 31(2) of the Children Act 1989 contains the harm threshold for triage:

‘A court may only make a care order or supervision order if it is satisfied.

- That the child concerned is suffering, or is likely to suffer, significant harm; and.
- That the harm, or likelihood of harm, is attributable to.
  (i) The care given to the child, or likely to be given to him if an order were not made, not being what it would be reasonable to expect a parent to give to him; or.
  (ii) The child being beyond parental control.’

The Act explains in Sect. 31(9) that: ‘health means physical or mental health; and development means physical, intellectual, emotional, social or behavioural development’.

Section 31(10) indicates but does not define what level of harm crosses the threshold:

‘Where the question of whether harm suffered by a child is significant turns on.

the child’s health and development, his health or development shall be compared.

with that which could reasonably be expected of a similar child’.

Three factors in the above definition of the harm threshold are salient to decisions about medical treatment for children; ‘significant harm’, ‘reasonable parent’ and ‘similar child’ and are below compared with the approach in Dutch and German law.

### Significant Harm

Neither Dutch nor German statutory law offers a description of ‘significant harm’. The courts in the three jurisdictions however *accept* that children may be disadvantaged by parental decisions. For England & Wales’ Mr Justice Hedley (as he then was) stated:

‘[…], that society must be willing to tolerate very diverse standards of parenting, including the eccentric, the barely adequate and the inconsistent. It follows too that children will inevitably have both very different experiences of parenting and very unequal consequences owing from it.’ [58]

is echoed by the German Federal Constitutional Court^1^:

‘It is not the task of the State to ensure an optimal development of the capabilities of the child against the will of the parents. The constitution has left the power of decision-making with regards to their child to the parents. It is accepted that children may be disadvantaged due to the decisions of their parents’ [17]

The three jurisdictions thus agree that the significance of harm is to be determined in court based on the specifics of each case. However, which factors can be taken into account in that determination differs across the jurisdictions. Similar to the courts in England & Wales [60], German law allows for a consideration of wider harm than merely medical considerations, namely ‘the physical, mental or spiritual well-being of the child’ [67]. In contrast, the Dutch harm threshold, likely due to its placement in the Health Care Act *only* allows for considerations about the health of the child to be taken into account [20]. The factors that courts can take into account in assessing whether the harm threshold is crossed are important because they determine the outcome in individual cases.

### Parental Decision-Making

The three jurisdictions agree that the actual or future harm must be due to parental decision-making but again do so differently. The German law speaks of parents that are either ‘not willing or not able to avoid the harm’ [67]. Similarly Dutch law focusses on the harm to be avoided and merely states that a parental refusal of medical treatment that is necessary to avoid significant harm to the child can be overruled by the court [20]. In contrast, the Children Act speaks of parental care that must not fall below the standard of that of a ‘reasonable parent’. This has been interpreted in court as an *objective* standard of parental care [41]. The focus of English judges is thus not *exclusively* on the significance of harm but also evaluates parental decision-making.

### Similar Child

Neither Dutch nor German law explicitly considers the child in question whereas the Children Act directs the judge to compare the child to a ‘similar child’ in order to determine whether the harm is significant. Judges have interpreted this clause to mean a child with similar attributes such as sex, age and ethnic origin. For example, Munby J commented: ‘the court must always be sensitive to the cultural, social and religious circumstances of the particular child and family’ [2]. Not all attributes of children can be compared but in medical treatment decisions we can expect the child to be compared to a child with a similar health condition.

In summary, the harm thresholds in the three jurisdictions are similar with regards to the opacity of the term ‘significant harm’ but differ in which considerations judges can take into account in the determination whether it is crossed. In addition, whilst Dutch and German law focusses on the significance of harm, the law in England & Wales also contains an objective standard for parental care.

## Case Law Analysis

The case law analysis is divided in two parts. First I will investigate the effects of the current harm threshold for triage by analysing the cases about medical treatment in the context of S31 orders reported in England & Wales. This is followed by a comparison of case law in England & Wales with Dutch and German case law in order to distil characteristics of cases that might be denied a court hearing in England & Wales after introducing the harm threshold for triage. The effects of the introduction of a harm threshold as standard for decision-making, thus relating to *outcome* of individual cases is also investigated by comparing national case law with international case law. In both analyses it is assumed that the current harm threshold in the Children Act will be introduced in England & Wales.

### Compared Cases

The search identified eight judgments in which courts decided about medical treatment within the context of a S31 order. Details of the cases are summarised in Table 1 in the supplementary data. The search further identified 83 cases in England & Wales using the best interests test. To allow comparison this analysis includes only reported cases in which a parental refusal of proposed medical treatment is litigated. All included cases are summarised in Table 2 in the supplementary data.

As Table 2. shows 25 cases were heard in England & Wales, 13 in the Netherlands and 10 in Germany. No conclusions can be drawn about the frequency with which these cases are heard in the courts in the respective jurisdictions on the basis of these numbers; especially in Germany district court cases are seldom reported. Unfortunately, the three jurisdictions do not report the actual number of court decisions about medical treatment for children.

### Harm Threshold for Triage in England & Wales

As mentioned above, the question to be answered in triage is whether the case can be decided in court. A case that does not cross the harm threshold leaves the decision to those with parental responsibility. Importantly, jurisprudence about the application of the harm threshold in the Children Act has developed in the context of child protection. Below I discuss relevant case law determining the application of the harm threshold and the identified S31 orders about medical treatment in more detail. Two factors were found to be important.

#### *Procedure*

In S31 orders it is the task of the applicant, in all identified cases the Local Authority, to prove, on the balance of probability [59], that the harm threshold is crossed.

To do so the Local Authority submits a ‘threshold document’ to the court in which they set out their evidence. For an applicant it is advantageous to present as much evidence as possible in order to maximise the chance that the harm threshold is considered crossed. Due to the objective standard against which parental care is measured there is a focus on parental behaviour and characteristics that can be presented as parental failings. In the included cases Local Authorities have presented relatively trivial evidence i.e. missed medical appointments [52, 57] and previous occasions when parents did not follow medical advice [3, 56, 57] as well as more serious concerns such as ‘inappropriate’ behaviour towards healthcare professionals [56], evidence about parental abilities and/or mental health [14, 42, 46, 53, 57] and parental personal history and relationships [14, 53, 57]. This scrutiny of parental failings is lacking in court decisions about medical treatment using a best interests test; whilst parental *reasons* are scrutinised, parental failings are not. Due to the emphasis on parental failings the content of the threshold document can be experienced as both intrusive and adversarial which may negatively impact future relationships between the family and the clinical team when the applicant is a NHS Trust.

#### *The Role of Medical Evidence*

In order to decide whether the harm threshold is crossed judges must first establish the facts. In decisions about medical treatment the medical evidence about the health condition of the child and the benefits/harms of proposed treatment is crucial in the establishment of those facts. Whilst the traditional deference of the court to medical experts may have abated to some extent [15, 62] it is still undeniably true that doctors are in a much better position to provide medical evidence than parents. More so as the decision that the harm threshold is crossed is accepted to be a value judgement [61]. Indeed, in all identified cases the medical evidence was accepted and thus the harm threshold considered crossed. That includes decisions in which the medical evidence is an opinion rather than fact-based. In *Re R*[57] for examplethe parents preferred to tube-feed their child by blending regular food (blended diet) rather than using commercially prepared feeds. Despite a lack of evidence that a blended diet is inferior, the judge decided the child should be fed with commercially prepared feeds given the preference of the treating doctor. The same importance to medical opinion is attached when establishing future harm. To establish future harm the applicant has to show that future harm is a ‘real possibility’ [61]. In M-W (a child) [46] an appeal court directed a case to be re-listed in High Court, to enable a medical opinion regarding the child’s future psychological and emotional development on the basis of maternal characteristics to be taken into account when no harm was demonstrable at the time of the ruling. The approach was confirmed by the Supreme Court a few years later when, in the absence of demonstrable harm, a care and adoption order was approved against parental wishes based on parental characteristics only [55].

In summary, in applying the harm threshold for medical treatment decisions in S31 orders factors come into play that are not present in decisions using the best interests test. In the context of S31 orders the threshold decision also takes into account parental characteristics and behaviour interpretable as parental failings resulting in a more adversarial procedure. In addition, medical evidence plays a crucial role in the determination whether the harm threshold is crossed also when it is based on opinion rather than scientific facts, putting parents at a considerable disadvantage.

### Harm Threshold for Triage in Medical Treatment Decisions

For decisions about children the courts in England & Wales currently use a best interests test for triage. In this section I investigate 1) whether introducing the harm threshold will deny cases access to court and 2) if so, the characteristics of those cases.

#### *Goal of Medical Treatment: Saving Life*

That the harm threshold will be crossed and the case thus heard in court seems all but certain when the child’s life is at stake. That is important because medical treatment intended to prevent loss of life is the topic of 70% of court decisions across the jurisdictions (32/46) and 85% (21/25) in England & Wales. However, in establishing whether the harm threshold is crossed judges must also take into account the *likelihood* that medical treatment prevents death. Refusing treatment that has little chance of avoiding the death of the child should not cross the harm threshold. Two such cases have been decided in England & Wales which I will discuss in more detail below.

In the first case the proposed treatment was experimental and had an estimated chance of curing the child’s leukaemia of 10% [29]. The case was brought to court because the parents disagreed; the mother favoured treatment given its life-saving potential, the father declined because of its burdens. In view of the low likelihood of cure and reasonable arguments of both parents in future similar cases might not cross the harm threshold and thus denied a court decision. However, when parents cannot agree and the issue is a serious one, there is an expectation that clinicians apply to the court for a decision [5]. Should the introduction of the harm threshold for triage deny cases with limited harm and reasonable but disagreeing parents a court hearing, those parents might be forced to litigate against each other instead.

In the second case the NHS Trust requested the court’s consent for surgery to enable continuation of haemodialysis for a child with kidney failure. The clinicians agreed that the choice between active treatment and palliative care for this particular child was evenly balanced [44]. A parental decision to refuse consent would thus seem reasonable. However, the parents based their refusal exclusively on their religious views, favouring prayer therapy instead. Likely, the clinicians’ unease about parental decision-making led to the court application. For now it remains an open question whether a parental preference for unproven treatment would fall below the standard of ‘reasonable parent’ when proven treatment has limited chance of success. Should in future such cases be denied access to the court an application contesting parental capacity might be made in some cases.

#### *Goal of Medical Treatment: Avoiding Harm*

In 16 cases across the jurisdictions and 4 in England & Wales consent was sought from the court to allow or prohibit non-life saving treatment.

In all three jurisdictions cases in which treatment sought to prevent the loss of vision or hearing have been litigated, including in a S31 order [14]. Similarly, cases about proposed medical treatment for children with severe mental health issues have been heard in Germany, the Netherlands and in a S31 order [62]. Such cases will thus continue to be heard in England & Wales should the harm threshold for triage be introduced.

However, harm to the child is not always imminent. In 6 cases litigated in the Netherlands and Germany a variety of medical treatment was proposed aiming at preventing harm (mostly) experienced in adulthood such as short stature and cardio-vascular disease. Should similar cases arise in England & Wales the harm threshold in itself should not deny such cases a court hearing.

In one of the remaining two cases about non-life saving treatment decided in England & Wales, the parents did not accept the diagnosis of incurable cancer and therefore palliative treatment [7]. Given that the diagnosis was well established the parental reasons for their refusal to consent might fall below the standard of reasonable parents. Moreover, the child was in considerable pain. Taken together, this case would have crossed the harm threshold and similar cases can be expected to be heard in court.

The second case is more controversial. The parents refused to consent to a brainstem test, to determine whether the child was legally dead, as they feared that the test might further injure their child [11]. The child was thought by clinicians to have lost the capacity to suffer and thus would not suffer himself from continued intensive care treatment. Under those circumstances it seems possible and perhaps even likely that the harm to the child posed by continued medical treatment will not be significant enough to cross the harm threshold and similar cases might not be heard in court.

In summary, after an introduction of the harm threshold for triage most cases about life-saving treatment will continue to be heard in the courts of England & Wales. A likely exception are cases in which the chance of successful treatment is low. So far such cases are rare and only lead to court applications when there are concerns about parental decision-making. Most cases litigating non-life saving treatment would also still be heard given the extent of potential harm in cases litigated so far. However, cases in which the child is no longer capable of suffering may be denied a court hearing.

### Harm Threshold as Standard for Decision-Making

In this section I compare the *outcome* of cases when either the harm threshold or the best interests test is used as standard for decision-making.

#### *Goal of Medical Treatment; Saving Life*

In 30/32 cases the courts provided substituted consent for proposed medical treatment intended to be life-saving. In more than 30 years of litigation across the three jurisdictions only twice did courts approve parental refusal of life-saving treatment.

In *Re T*[54], the appeal court in England & Wales, decided it was not in the child’s best interests to have a life-saving liver transplant. In the specific circumstances the transplant would involve the mother being forced to stay in England to care for a child with substantial medical needs due to a procedure she opposed given the burdens of treatment, whilst the father lived and worked abroad. *Re T* does however seem to be an outlier as equally invasive treatment also requiring considerable familial input, e.g. a bone marrow transplant, has since been allowed against the wishes of the parents [49]. Similarly, courts in Germany and the Netherlands have authorised intensive treatment, i.e. chemotherapy demonstrating that a harm threshold does not prohibit intensive medical treatment against parental wishes when potentially life-saving.

In the second case a 4-year old German child in ‘Wachkoma’^2^ but suffering from severe, painful muscle spasms following a hypoxic incident resided in a rehabilitation centre. Given that the child was not expected to regain awareness and the painful symptoms could not be relieved except by deep sedation the parents decided stop her clinically-assisted feed and allow her to die at home under the supervision of a palliative care specialist. Her doctors disagreed. The lower court declared the harm threshold crossed [4]. However, the appeal court found that given the prognosis and the inability to treat her pain other than by sedation the foreseeable death of this particular child did not cross the harm threshold [50].

The above demonstrates that, regardless of the standard used, for parents to obtain the court’s approval of their refusal of life-saving medical treatment is a very high bar indeed. In the two cases in which the death of the child was allowed, the exceptional circumstances determined the court’s ultimate decision. The second case also demonstrates that health care professionals are uncomfortable when parents decide, ahead of them, that their child should be allowed to die. Such cases have been discussed in the literature [16, 64] and in England & Wales has been the subject of a S31 order [57].

#### *Goal of Medical Treatment; Avoiding Harm*

In most cases, the courts also allow medical treatment that is not intended to be life saving. In 12/14 cases (85%) in which parents refused proposed medical treatment not intended to be life-saving, the courts provided substituted consent. Both cases in which the court declined to do so were decided using a harm threshold.

In Germany deaf parents refused the implantation of a cochleair device in their youngest child [6]. In a reasoning very similar to that in *Re T*[54] the German court balanced the impact on family life, the child spending many hours outside the family home in order to acquire spoken language after implantation of the device, against the harm of not being able to hear and concluded that the harm threshold was not crossed. The case demonstrates that a court decision using a harm threshold can be equally holistic as when using a best interests test. However, whether a holistic approach is taken does depend on how the harm threshold is defined. Should the German case described above have been heard in the Netherlands the court might well have provided substituted consent for cochleair implantation because the Dutch harm threshold only allows consideration of the *health* of the child. Moreover, that factors *can* be taken into account does not mean that they *will* be taken into account. For example, in circumstances in which parents were thought not to be able to afford the implantation of a cochleair device a court in England allowed a care and adoption order [14] against their wishes. The judgment takes into account medical evidence but not the potential harm caused by the child losing contact with her birth family.

In the second case, a Dutch court was asked to allow a renal transplant for a child suffering from chronic renal failure [23]. Whilst the child’s health was stable on dialysis, the main aim of the transplant was to prevent serious cardio-vascular problems in adulthood caused by metabolic dysregulation inherent to renal failure. Because the harm would be experienced in adulthood and treatment could be deferred to when the child could decide the court considered the harm threshold not crossed. Given that part of the harm does occur in childhood it is likely that the best interests test would have allowed the renal transplant to go ahead in childhood. The argument that a decision can be deferred until children can decide for themselves has also been used in Germany to prohibit cross-sex hormone treatment for gender dysphoria. However, deferring treatment until the child can decide may be less convincing to the courts in England & Wales. In 2013 a court approved booster immunisation against mumps, measles and rubella (MMR) for a healthy 15-year old against her wishes [24]; a decision that could have been deferred until she reached adulthood with minimal or no harm.

In summary, in the vast majority of cases in which parents and clinicians disagree about proposed treatment the outcome is similar regardless of the standard used in court. In most cases the courts provide substituted consent. The similar outcomes are likely due to the severity of preventable harm in the cases heard in the courts across the three jurisdictions.

Taken together, the results indicate that introducing the harm threshold either for triage or as standard for decision-making will not substantially limit the number of future court applications. Nevertheless, the investigation found three characteristics of cases for which litigation might change. When introduced for triage two types of case could be denied a court hearing; 1) cases in which harm is limited and there are concerns about parental decision-making and 2) when parents refuse treatment for a child not capable of suffering. Only under these specific circumstances will the introduction of the harm threshold likely increase parental discretion. An introduction of the harm threshold as standard might change the outcome of cases when treatment can be deferred to adulthood increasing the autonomy of the child rather than their parents.

Parental discretion might be further increased should a harm threshold be introduced that does not contain an objective standard of parental care. An introduction of a harm threshold for medical treatment decisions that directs the courts to focus on the significance of harm only might change the outcome of cases in which the harm is limited. For example, in the Netherlands a mother based the refusal of a renal transplant on her belief that her child would be miraculously cured [23]. In England & Wales it is not uncommon for parents to place their faith in a ‘miracle cure’ [48] or have other faith-based reasons [45] which currently are unlikely to take precedence over medical evidence. A harm threshold that only takes into account the harm and not parental reasoning may go some way to broaden parental discretion when harm is limited.

## Withholding and Withdrawing Treatment

So far, this investigation evaluated decisions in which parents *refuse* medical treatment for their child. In the next section I attempt to evaluate what the effects of the introduction of the harm threshold might be for cases about withholding and withdrawing life-sustaining treatment.

### Harm Threshold for Triage

The investigation has demonstrated that cases that may be denied a court decision are those in which the harm ascribed to parental decision-making is limited and in which the child does not suffer. A common argument in decisions about withholding and withdrawing medical treatment applying is that the child is or would suffer treatment-related harm. However, in some recent cases it is accepted that the child does not have the capacity for suffering [1, 9, 11, 39]. In the absence of suffering the harm done to the child by continuing treatment may be too insufficient to cross the harm threshold and might thus be denied a court hearing.

### Harm Threshold as Standard for Decision-Making

A substantive decision using a harm threshold as standard would involve a more holistic approach than when used for triage. Arguments could include complex concepts such as dignity and balance benefits and harms of continuing life against death.

The question whether a child can suffer when she is in a persistent vegetative state has recently been discussed in the case of Pippa Knight [33]. The legal team representing the mother argued that medical treatment did not harm the child because she did not have the capacity to suffer. The judge found that the daily treatment-related interventions were nevertheless burdens on the child that should be taken into account [32]. The outcome of such considerations depend on the balance between level of awareness and severity of treatment burden. In the case of Tafida Raqeeb, a child needing mechanical ventilation but who was otherwise stable, MacDonald found that the child’s minimal awareness was sufficient to continue medical treatment given the low burdens thereof [8].

It has been suggested that loss of dignity should be considered a harm when continuing medical treatment is futile [65]. Indeed, ‘dying with dignity’ is sometimes referred to in judgements about withdrawal of treatment [26]. However, so far judges have declined to define the burden of (futile) medical treatment in terms of dignity because of its subjectivity [10, 33]. If the concept of dignity is not taken into account, suffering and thus harm will be defined by the balance between burden of treatment and benefits of continued life as it is now. The introduction of the harm threshold as standard for decision-making should not substantially change these decisions.

## Conclusion

This article investigated the effects of an introduction of the harm threshold, either for triage or as standard for medical treatment decisions for children in the courts of England & Wales. By analysing and comparing legal context, national and Dutch and German case law I found that an introduction of the harm threshold similar to the one in the Children Act will not broaden parental discretion or reduce the amount of litigation but will introduce new challenges. Two factors are important drivers of this conclusion; the extent of harm in cases that currently reach the courts and the crucial role of medical evidence in establishing the relevant facts of the case.

When introduced as standard for decision-making the outcome of cases can be expected to be by and large the same. However, when introduced for triage, the harm threshold likely excludes cases from court in which (1) harm is limited because the treatment is unlikely to succeed or (2) the child has lost the capacity to suffer. In the first case the effect will be minimal as such cases are very rare and only litigated when there are additional concerns about parental decision-making. However, when the child is no longer capable of suffering the harm threshold used for triage could prevent cases reaching the courts. Applied to cases in which withdrawing life-sustaining treatment is the topic of litigation may lead to more severely compromised children continuing life-sustaining treatment when that treatment could be considered futile.

The investigation leaves open the question which legislative steps *would* increase parental discretion for medical treatment decisions in England & Wales. It may be useful to first attempt to reach consensus about the specific circumstances in which broader parental discretion is desired before specific legislative steps are considered. Currently, it is argued that parents should be the decision-makers when alternative treatment is available [65] or should be allowed a period of time to arrange alternative treatment when the court allows withdrawal of life-sustaining treatment against their wishes [51] but consensus on either proposal has yet to be reached [47]. Notably, the introduction of the current harm threshold does not guarantee broader parental discretion in the first circumstance as the decision will depend on the factors taken into account. In the case of Charlie Gard for example a court decision may have turned on the question whether the side effects of the treatment proposed by the parents should have been added to the already existing burden of treatment due to the mechanical ventilation or seen in isolation. When added to the existing burden the harm threshold might have been crossed.

In conclusion, an introduction of the harm threshold, either for triage or as standard for decision-making is unlikely to substantially increase parental discretion other than under very specific circumstances and thus will not reduce the number of cases litigated in the courts of England & Wales.

## Supplementary Information

Below is the link to the electronic supplementary material.

Supplementary file1 (DOCX 12 kb)

## Data Availability

The source data for this investigation is public as described in the Methods section.

## Footnotes

1 The Federal Constitutional Court is the highest constitutional court in Germany.

2 A condition previously known as Persistent Vegetative State. The current medical term is Prolonged Disorder of Consiousness.
