# Supplementary material for: The Effects of Introducing a Harm Threshold for Medical Treatment Decisions for Children in the Courts of England & Wales: An (Inter)National Case Law Analysis
Source: Health Care Anal. 2023 Dec 18;32(3):243–59. doi: 10.1007/s10728-023-00472-w (PMC11390764; doi:10.1007/s10728-023-00472-w)
Supplement: Supplementary file 1 — Supplementary file1 (DOCX 12 kb) [file 10728_2023_472_MOESM1_ESM.docx]

Table 1: Medical treatment decisions for children in the context of S31 orders

|  | **Name** | **Medical condition** | **Outcome** | **Factors contributing to crossing of harm threshold** |
| --- | --- | --- | --- | --- |
| 1 | *Re O (care or supervision order)* [1996] 2 FLR 755 | General health issues (6 children) | Threshold conceded | 1. Missed medical appointments  2. Weight loss due to diet  3. Physical and cognitive developmental delay  4. For one child avoidable loss of vision in one eye. |
| 2 | *Re v (Care or supervision order)* [1996] 1 FLR 776 | Severe Cerebral Palsy  Learning disability | Threshold crossed | Need for:  1. Physiotherapy for improvement of mobility, posture and development  2. Sufficient stimulation for emotional/intellectual development |
| 3 | *M-W (a child)* [2010] EWCA Civ 12 | Prematurely born, complex needs including a tracheotomy for laryngeal web, fed by naso-gastric tube, chronic lung disease needing overnight extra oxygen | Re-hearing ordered | Medical opinion re potential future harm in casu emotional development due to maternal mental health issues should be taken into account in the determination whether harm threshold is crossed |
| 4 | *A Local Authority and a NHS Trust v MC and FC* [2017] EWHC 370 (Fam) | Prematurely born, severe cerebral palsy, disordered swallowing risking chest infections due to aspiration of food, restricted lung function and frequent chest infections.  Life-limiting disorder* | Threshold crossed | Unsafe maternal feeding practice risking chest infections due to aspiration and malnourishment |
| 5 | *Re AB (a child) (Care Proceedings: medical treatment)*[2018] EWFC 3 | Neuro-metabolic disorder | In 1^st^ instance threshold crossed** | Child suffering unnecessary pain due to parents  1. Lowering dose of rescue medication  2. Removing care plan from bedroom  3. Difficult working relationship with healthcare professionals |
| 6 | *Re R (a Child)* [2018] EWFC 28 | Rett syndrome | Threshold crossed | Due to maternal mental health issues  1. Missed medical appointments  2. Refusal to follow medical advice  3. Inappropriate refusal of antibiotics for chest infections and possible refusal of hospital care in health crisis |
| 7 | *London Borough of Barking and Dagenham v A* [2019] EWHC 2017 (Fam) | Severe mental health  issues | Threshold crossed | 1. Complex needs that can only be met by residence in therapeutic setting  2. Mother vulnerable due to learning disabilities |
| 8 | *Birmingham City Council v SQ and others* [2019] EWHC 850 (Fam) | 1. Profoundly deaf  2. Developmental delay | Threshold crossed  for care and adoption order | 1. Medical treatment of deafness by implantation of cochleair device needed  2. Mother assessed as not capable of looking after child on her own due to vulnerabilities thus child in foster care whilst parents in Pakistan  3. Alternative Pakistani family carers not granted visa  4. Medical treatment not possible in Pakistan due to financial situation parents  5. Difficult working relationship between family and local authority |

* Case heard in combination with NHS Trust application for ceiling of care. Future hearing ordered to discuss evidence, in interim ceiling of care allowed.

** On appeal application for care order withdrawn by local authority

Table 2: Details of cases used in the comparison of (inter)national case law

|  | **Name** | **Country** | **Medical treatment** | **Intention of treatment** | **Treatment allowed** |
| --- | --- | --- | --- | --- | --- |
| 1 | *Re S (a minor)(medical treatment)*[1993] 1 FLR 376 | England & Wales | Blood transfusion | Life saving | Yes |
| 2 | *Re O (a minor)(medical treatment)*[1993] 2 FLR 149 | England & Wales | Blood transfusion | Life saving | Yes |
| 3 | *Re R (a minor)(blood transfusion)* [1993] 2 FLR 757 | England & Wales | Blood transfusion | Life saving | Yes |
| 4 | *Re E (A minor) (Wardship: medical treatment)* [1993] 1 FLR 386 | England & Wales | Blood transfusion | Life saving | Yes |
| 5 | *Re T (a minor) (wardship: medical treatment)* [1997] 1 All ER 906 | England & Wales | Liver transplant | Life saving | No |
| 6 | *Re C (a child)(HIV test)* 50 BMLR 283 | England & Wales | HIV test | Life prolonging | Yes |
| 7 | *Re A (children) (conjoined twins)* [2000] EWCA Civ 254 | England & Wales | Surgical separation | Life saving for one twin | Yes |
| 8 | *NHS Trust v A* [2007] EWHC 1696 (Fam) | England & Wales | Bone marrow transplant | Life saving | Yes |
| 9 | *An NHS Trust v SR [2012] EWHC 3842* (Fam) | England & Wales | Conventional cancer treatment | Life saving | Yes |
| 10 | *Birmingham Children’s NHS Trust v B & C* [2014] EWHC 531 (Fam) | England & Wales | Blood transfusion | Life saving | Yes |
| 11 | *An NHS Trust v Child B and others* [2014] EWHC 3486 (Fam) | England & Wales | Blood transfusion | Life saving | Yes |
| 12 | *Re AA* [2015] EWHC 1178 (Fam) | England & Wales | Implantation pacemaker | Life saving | Yes |
| 13 | *Re JM (a child)* [2015] EWHC 2832 (Fam) | England & Wales | Conventional cancer treatment | Life saving | Yes |
| 14 | *NHS Foundation Trust v T and another* [2016] EWHC 2980 (Fam) | England & Wales | Platelet transfusion | Life saving | Yes |
| 15 | *Re EQ* [2016] EWHC 3418 (Fam) | England & Wales | Cataract surgery | Prevention loss of sight | Yes |
| 16 | *An NHS Trust v BK and others* [2016] EWHC 2860 (Fam) | England & Wales | Palliative care | Palliation | Yes |
| 17 | *Manchester University Hospital NHS Foundation Trust v M* [2019] EWHC 468 (Fam) | England & Wales | Insertion catheter for dialysis | Life saving | Yes |
| 18 | *Cardiff and Vale University Health Board v T (a minor) and another* [2019] EWHC 1671 (Fam) | England & Wales | Blood transfusion | Life saving | Yes |
| 19 | *Re X* [2020] EWHC 1630 (Fam) | England & Wales | Blood transfusion | Life saving | Yes |
| 20 | *Re X* [2020] EWHC 3003 (Fam) | England & Wales | Blood transfusion | Life saving | Yes |
| 21 | *An NHS Foundation Trust v AB* [2020] EWHC 3221 (Fam) | England & Wales | Cataract surgery | Prevention loss of sight | Yes |
| 22 | *Cambridge University Hospital NHS Foundation Trust v E and others* [2021] EWHC 126 (Fam) | England & Wales | Conventional cancer treatment | Life saving | Yes |
| 23 | *Re A* [2021] EWHC 2517 (Fam) | England & Wales | Experimental cancer treatment | Life saving | Yes |
| 24 | *Re ZY* [2022] EWHC 1328 (Fam) | England & Wales | General anaesthetic for biopsy (suspected cancer) | Potentially life saving (if cancer diagnosis confirmed) | Yes |
| 25 | *Barts Health NHS Trst v Dance and others* [2022] EWHC 1165 (Fam) | England & Wales | Brainstem test | Diagnostic | Yes |
| 26 | ECLI:NL:GHSGR:1999:AB0949 | Netherlands | Blood transfusion | Life saving | Yes |
| 27 | ECLI:NL:RBSGR:2002:AO5311 | Netherlands | Blood transfusion | Life saving | Yes |
| 28 | ECLI:NL:RBUTR:2009:BI7161 | Netherlands | Occlusion therapy | Prevention loss of sight | Yes |
| 29 | ECLI:NL:RBUTR:2010:BN6672 | Netherlands | Kidney transplant | Prevention cardio-vascular disease in adulthood | No |
| 30 | ECLI:NL:RBAMS:2010:BL9136 | Netherlands | Blood transfusion | Life saving | Yes |
| 31 | ECLI:NL:RBALM:2010:BO9506 | Netherlands | Conventional cancer treatment | Life saving | Yes |
| 32 | ECLI:NL:RBARN:2012:BY7699 | Netherlands | Blood transfusion | Life saving | Yes |
| 33 | ECLI:NL:RBOVE:2016:2221 | Netherlands | Mental health treatment | Management non-fatal condition | Yes |
| 34 | ECLI:NL:RBZWB:2016:7795 | Netherlands | Blood transfusion | Life saving | Yes |
| 35 | ECLI:NL:RBROT:2020:1858 | Netherlands | Blood transfusion | Life saving | Yes |
| 36 | ECLI:NL:RBOBR:2020:3694 | Netherlands | Hospital-based treatment prematurity/  neonatal abstinence syndrome | Management non-fatal condition | Yes |
| 37 | ECLI:NL:RBROT:2020:10737 | Netherlands | Blood transfusion | Life saving | Yes |
| 38 | ECLI:NL:GHSHE:2021:2780 | Netherlands | Administration growth hormone and puberty blockers | Prevention short stature | Yes |
| 39 | OLG Celle – 21.02.1994 – 17 W 8/94 | Germany | Blood transfusion | Life saving | Yes |
| 40 | BayObLG – 22.03.1995 (1Z BR 120/94) | Germany | Rehabilitation after hip surgery for disabled child | Management non-fatal condition | Yes |
| 41 | OLG Stuttgart Beschluß v. 06.12.2001 – 17 UF 377/01 | Germany | Psychosocial growth deficit | Prevention short stature | Yes |
| 42 | OLG Brandenburg 04.08.2003 9 WF 117/03 ( WF 118/03 | Germany | Anorexia nervosa/Diabetes mellitus type I | Life saving | Yes |
| 43 | Amtsgericht Minden, 32 F 53/07 (ECLI:DE:AGMI1:2007:0313.32F53.07.00)  OLG Hamm, 24.05.2007 – 1 UF 78/07 | Germany | Withdrawal artificial feeding | Life saving | No |
| 44 | Brandenburgisches Oberlandesgericht 29.10.13 – 13 UF 208/13 | Germany | Treatment mental health issues | Management non-fatal condition | Yes |
| 45 | Amtsgerichts Wetzlar vom 10.2.2015  OLG Frankfurt/Main, Beschluss vom 04.02.2016 - Aktenzeichen 4 UF 274/15 | Germany | Treatment malnutrition | Prevention permanent in childhood/  adulthood | Yes |
| 46 | AG Goslar 28.01.2019 – 12 F 226/17 SO | Germany | Cochleair implantation | Prevention loss of hearing | No |
